# Roles of the crucial mitochondrial DNA in hypertrophic cardiomyopathy prognosis and diagnosis: A review

**DOI:** 10.1097/MD.0000000000036368

**Published:** 2023-12-01

**Authors:** Xuewen Liao, Shunkai Zhou, Dehua Zeng, Wenmin Ying, Duohuang Lian, Meiqing Zhang, Jianjun Ge, Mengmeng Chen, Yaming Liu, Yazhou Lin

**Affiliations:** a Department of Cardiology, Fujian Provincial Hospital, Fuzhou City, China; b Department of Thoracic and Cardiac Surgery, 900th Hospital of the Joint Logistics Support Force of the Chinese People’s Liberation Army, Fuzhou City, China; c Department of Pathology, 900th Hospital of the Joint Logistics Support Force of the Chinese People’s Liberation Army, Fuzhou City, China; d Department of Radiotherapy, Fuding Hospital, Fuding City, China; e Department of Thoracic Surgery, No. 2 Hospital of Nanping City, Nanping City, China.

**Keywords:** hypertrophic cardiomyopathy, immune cells, mitochondrial DNA

## Abstract

Mitochondrial DNA is implicated in hypertrophic cardiomyopathy (HCM) development. We aimed to identify valuable mtDNAs that contribute to the development of HCM. Differentially expressed mitochondrial DNAs (DEMGs) between HCM and controls were screened. GO and KEGG functional enrichment analyses were performed, and the optimum genes were explored using the LASSO regression mode and SVM-RFE model. A diagnostic scoring model was constructed and verified using ROC curves. Mitochondria-based subtypes were identified. Immune performance among the subtypes including immune cells, immune checkpoint genes, and HLA family genes was analyzed. Finally, an mRNA-transcription factor (TF)-miRNA network was constructed using Cytoscape software. Twelve DEMGs in HCM were selected. Among them, 6 DEMGs, including *PDK4, MGST1, TOMM40, LYPLAL1, GATM*, and *CPT1B* were demonstrated as DEMGs at the point of intersection of Lasso regression and SVM-RFE. The ROC of the model for the training and validation datasets was 0.999 and 0.958, respectively. Two clusters were divided, and 4 immune cell types were significantly different between the 2 clusters, including resting mast cells, macrophages M2, and plasma cells. Nine upregulated KEGG pathways were enriched in cluster 1 vs. cluster 2 including O-glycan biosynthesis, the ErbB signaling pathway, and the GnRH signaling pathway. Meanwhile, 49 down-regulated pathways were enriched such as the toll-like signaling pathway and natural killer cell-mediated cytotoxicity pathway. The 6 gene-based mRNA-TF-miRNA networks included other 133 TFs and 18 miRNAs. Six DEMGs in HCM, including P*DK4, MGST1, TOMM40, LYPLAL1, GATM*, and *CPT1B*, can be indicative of HCM prognosis or disease progression.

**Highlights**

1. Valuable mtDNAs contributing to the development of HCM was investigated;
2. Six DEMGs were selected, including *PDK4, MGST1, TOMM40, LYPLAL1, GATM*, and *CPT1B*;
3. Immune cells, including mast cell resting, macrophages M2, and plasma cells play roles in HCM.

## 1. Introduction

Hypertrophic cardiomyopathy (HCM) is one of the most common inherited heart diseases worldwide, with a general prevalence of 1:500.^[1]^ Generally, this disease is characterized by left ventricular hypertrophy and reduced diastolic function. The etiology of HCM includes multiple factors including environmental toxins, neuromuscular disorders, and genetic factors.^[2]^ An identifiable mutation in a sarcomeric protein is the main etiology of HCM. In recent years, the role of various types of genetic mutations in the development of HCM have been confirmed. However, information on 40% of these cases still remains unclear.

Mitochondria are semi-autonomous organelles, which have their own genomes, replication machinery, and protein synthesis processes. It is reported that mitochondria play regulatory roles in cellular energy production,^[3]^ initiating apoptosis,^[4]^ and controlling calcium concentration.^[5]^ The crucial role of mitochondria in normal heart function has been verified, and mitochondrial dysfunction is highly related to HCM development. Accumulating evidence has determined that the mutations of mitochondrial DNA (mtDNA) are associated with various diseases, such as metabolic, degenerative diseases and cancer. The contributor of mtDNA variations in HCM has attracted considerable attention. Currently, multiple mtDNA mutations have been observed in HCM.^[6,7]^ Haplogroup T, defined as 13368A, was found to be significantly associated with HCM risk in Spanish patients with HCM.^[8]^ Mitochondrial dysfunction exerts a pathogenic role in HCM.^[9]^ Although the dominant role of mtDNA in HCM has been confirmed, little is known about the role of mitochondrial genes in the development and prognosis of HCM. Therefore, in the present study, GSE36961 and GSE141910 datasets were used as training and validation datasets, respectively, to screen out differentially expressed mitochondrial genes (DEMGs) between patients with HCM and healthy controls. Furthermore, optimum DEMGs related to HCM diagnosis were explored using the LASSO regression mode and SVM-RFE model. Furthermore, predicative activities of these genes and their related functional roles were explored. Finally, we identified 6 DEMGs in HCM, including PDK4, MGST1, TOMM40, LYPLAL1, GATM, and CPT1B, which might be important biomarkers for diagnosis or prognosis of HCM. Herein, we strengthened the causal role of mitochondrial dysfunction in HCM and pointed out a list of DEMGs of interest in HCM. Our findings may offer a promising avenue to understand the pathogenesis of HCM and guide the diagnosis and treatment of HCM in the future.

## 2. Material and methods

### 2.1. Data source

GSE36961 and GSE141910 datasets were downloaded from the NCBI Gene Expression Omnibus (http://www.ncbi.nlm.nih.gov/geo/).^[10]^ GSE36961 consisted of 145 samples, including 106 cardiac tissues from HCM and 39 control samples, which were sequenced using the GPL15389 platform (Illumina HumanHT-12 V3.0 expression beadchip). GSE141910 consisted of 194 samples, including 28 cardiac tissues from HCM and 166 control samples, which were sequenced using the GPL16791 platform (Illumina HiSeq 2500 [Homo sapiens]). In the present analysis, the GSE36961 dataset was used as the training set and GSE141910 was used as the validation dataset. The corresponding expression matrices for each dataset and the annotation file corresponding to the sequencing platform were downloaded. Furthermore, mitochondria-related genes were obtained from the human mitochondria-related database MitoCarta3.0 (https://www.broadinstitute.org/mitocarta/mitocarta30-inventory-mammalian-mitochondrial-proteins-and-pathways).^[11]^

### 2.2. Screen of DEMGs

Differentially expressed genes (DEGs) between patients with HCM and healthy controls were screened using the Limma package of R software (Version 3.10.3, http://www.bioconductor.org/packages/2.9/bioc/html/limma.html).^[12]^ The threshold was set as follows: *P* value < .05 &| logFC |>0.585. Next, Venn analysis was performed on the DEGs and mitochondrial genes to screen out DEMGs using the R package VennDiagram (version1.7.3).^[13]^

### 2.3. Functional analysis of DEMGs

To investigate the potential functions of DEMGs, gene ontology (GO) and Kyoto Encyclopedia of Genes and Genomes (KEGG) functional enrichment analyses were performed using the R package “clusterProfiler” (http://bioconductor.org/packages/release/bioc/html/clusterProfiler.html).^[14]^ Based on GO analysis, biological processes (BP), cellular components, and molecular functions (MF) associated with DEMGs were investigated.

### 2.4. Construction of protein-protein interaction (PPI) network

A PPI network for the DEMGs was constructed using STRING (version 11.5, http://www.string-db.org/).^[15]^ In the analysis, DEMGs were set as input genes, and the species was set as Homo sapiens. The threshold for PPI score was set as 0.15 (low confidence). Hub genes were selected based on the properties of nodes in the network.

### 2.5. Machine learning algorithm for identifying optimal genes

Diagnostic genes were explored using the LASSO regression mode and SVM-RFE model based on the expression values of DEMGs. LASSO regression model was constructed using R software glmnet package (version 4.1-6, httR iskscore://cran.r-project.org/web/packages/glmnet/index.html).^[16]^ The parameter settings were set as follows: family= “binary,” type. measure= “class,” nfolds = 10, which means 10-fold cross validation was performed to achieve LASSO logistic regression and screen out the characteristic genes that reach the lowest error rate.

SVM-Recursive Feature Elimination (RFE) model was constructed using the R package “e1071” (version 1.7-12, https://CRAN.R-project.org/package=e1071).^[17]^ Hub genes were sorted using an SVM algorithm. The importance and importance ranking of each gene were conducted using the RFE method. A combination with the lowest error rate was selected as anoptimal combination, and the corresponding genes were selected as characteristic genes for HCM.

Next, the regression coefficients of each diagnostic gene were calculated using LASSO regression, and the diagnostic score was calculated using the following formula:


$$\begin{array}{l} Risks\;score=\sum \beta Gene\times Expgene \end{array}$$

Here, β Gene represents the LASSO regression coefficient of the gene, while Expgene represents the expression level of the gene in each sample.

### 2.6. Evaluation and verification of the diagnostic scoring model

In the training dataset, ROC curves were plotted using R “pROC” (versions/1.18.0, https://www.expasy.org/resources/proc)^[18]^ after obtaining diagnostic scores. Meanwhile, RiskScore distribution of samples in different groups were also visualized using box plots.

The RiskScore calculation formula was used to verify the accuracy of the model, and the same regression coefficients were used to calculate RiskScore values for each sample in the validation dataset. RiskScore distributions were visualized using both receiver operating characteristic (ROC) curves and box plots.

### 2.7. Construction of PPI network

To predict the correlation between optimal DEMGs and their 20 interacting genes related to colocalization, shared protein structure domains, co-expression, prediction, and pathways, a PPI network was constructed using the GeneMANIA database (http://genemania.org/).^[19]^

### 2.8. Correlation between diagnostic genes and immunity

To observe the difference of immune microenvironment between normal and HCM samples, the CIBERSORT algorithm was used (http://cibersort.stanford.edu)^[20,21]^ to calculates the proportion of 22 immune cell components based on expression levels of the involved genes.

Furthermore, the Wilcoxon test was used to compare differences between normal and HCM groups, and Spearman correlation analysis was employed to analyze the correlation between diagnostic genes and differential immune cells.

### 2.9. Prediction of mitochondrial-based molecular subtypes

Mitochondrial-based molecular subtypes of patients with HCM were analyzed using ConsensusClusterPlus (Version 1.54.0, http://www.bioconductor.org/packages/release/bioc/html/ConsensusClusterPlus.html)^[22]^ based on expression levels of the diagnostic genes. The parameters were set as follows: number of bootstraps: 50; cluster algorithm: km; distance= “Euclidean”; item subsampling promotion: 0.8; feature subsampling promotion: 1.

### 2.10. Comparison of immune cells differences between DMEGs-based subtypes

To further explore the relationship between mitochondria-related molecular subtypes and the HCM immune microenvironment, the immune score and the score of 22 types of immune cells were determined employing ESTIMATE and CIBERSORT in R. The Wilcoxon test was used to evaluate differences in immune scores and immune cells among different subtypes.

### 2.11. Comparison of immune checkpoint genes and HLA family genes between DMEGs-based subtypes

Immune checkpoints refer to interactions that inhibit cytotoxic T lymphocyte (CTL) activation. Immune checkpoint molecules are crucial for immune function and have several clinical significances in immunotherapy. Therefore, we investigated correlation between HCM and the expression of 6 immune checkpoint molecules, including PDCD1 (PD1), CD274 (PDL1), PDCD1LG2 (PDL2), CTLA4, and CD80.

Expression data for immune checkpoint genes (such as PD1/PDCD1, PD-L1/CD274, and other genes) and HLA family genes were extracted. Next, the differences between immune checkpoint genes and HLA family genes among mitochondrial-based subtypes were compared using the Wilcoxon test.

### 2.12. GSEA enrichment of KEGG pathway between DMEGs-based subtypes

The MSigDB database v7.1 (http://software.broadinstitute.org/gsea/msigdb/index.jsp)^[23]^ combined with subtype information of the samples served as the enrichment background. To determine whether each KEGG pathway was significantly enriched in a certain subtype, GSEA analysis was carried out using the R packet clusterProfiler. After “BH” correction, the factor that displayed *P* < .05 was considered to be a significant enrichment result.

### 2.13. Transcription regulation analysis of optimal DMEGs

Transcription factors (TFs) regulate the expression of genes (TF). Hence, TFs targeting the selected optimal DMEGs were explored using the transcription factor database (http://bioinfo.life.hust.edu.cn/hTFtarget#!/).^[24]^ Moreover, the online database miRWalk3.0 (http://mirwalk.umm.uni-heidelberg.de)^[25]^ was used to predict key candidate genes related to miRNAs. MiRNA-mRNA relationship pairs were selected if the parameter binding probability was ≥ 0.8, and 3UTR was set as the binding site position. MiRNA-mRNA relationship pairs were selected when they were both selected using the TargetScan and miRDB databases. Next, the mRNA-TF-miRNA network was constructed using Cytoscape software (version 3.9.2).^[26]^

## 3. Results

### 3.1. DEMGs selected in HCM

**Figure 1.**
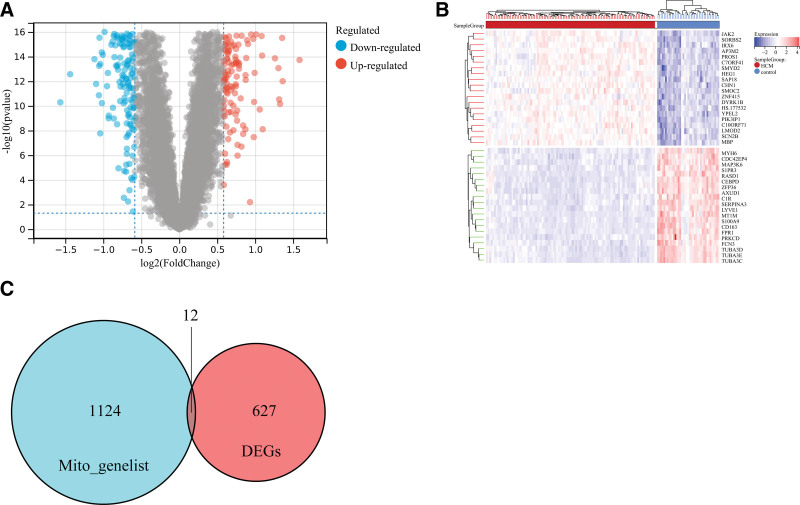
Volcano plot and heatmap of differentially expressed genes (DEGs) in hypertrophic cardiomyopathy (HCM), and venn diagram of differentially expressed mitochondrial genes (DEMGs) selection in HCM. A: Volcano plot of DEGs in HCM; B: Heatmap of DEGs in HCM; C: venn diagram of DEMGs selection in HCM.

In total, 639 DEGs, including 249 upregulated and 390 downregulated genes, were identified. The Volcano plot (Fig. 1A) and heatmap (Fig. 1B) of DEGs showed that the expression levels of DEGs in HCM were different from those in normal controls. A total of 12 DEMGs in HCM shown using the Venn plot (Fig. 1C) were selected.

### 3.2. Functional analysis of DEMGs and PPI construction

**Figure 2.**
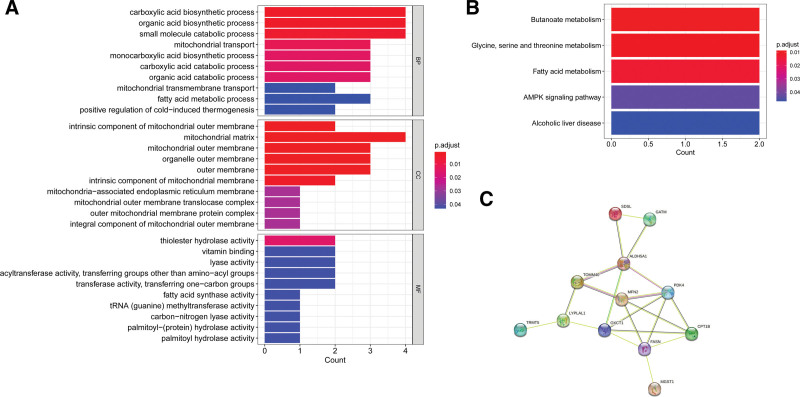
Functional enrichment and protein-protein interaction (PPI) network of differentially expressed mitochondrial genes (DEMGs) in hypertrophic cardiomyopathy (HCM). A: Top 30 gene ontology item enriched by DEMGs in HCM; B: Kyoto encylopaedia of genes and genomes enriched by DEMGs in HCM; C: PPI network of DEMGs in HCM.

The DEMGs were significantly enriched in 42 GO terms, including 17 BPs, 14 cellular components, and 11 MFs. The top 30 items are shown in Figure 2A, including the carboxylic acid biosynthetic process, organic acid biosynthetic process, and small molecule catabolic process. Moreover, DEMGs were significantly enriched in 5 KEGG pathways, including butanoate metabolism; glycine, serine, and threonine metabolism; and fatty acid metabolism (Fig. 2B).

As shown in Figure 2C, the PPI network included 20 interactive pairs. Among them, ALDH5A1, CPT1B, and FASN play a role as hub genes in the network.

### 3.3. Six DEMGs were selected as optimal diagnostic genes for HCM

**Figure 3.**
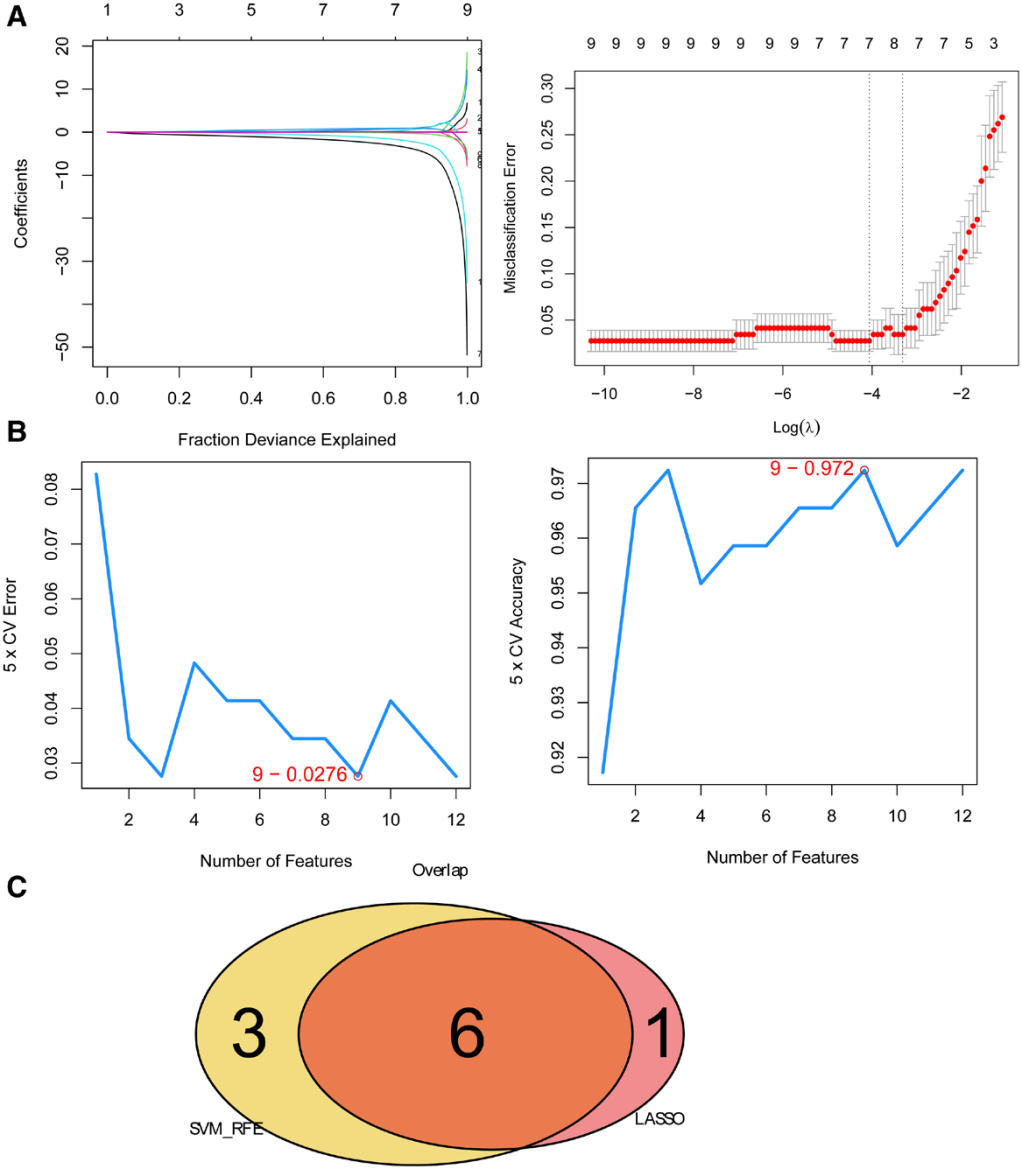
Machine learning algorithms for identifying key feature genes. A: Lasso regression analysis results; Left: LASSO model non-zero coefficient graph, each curve in the graph represents the trajectory of each independent variable coefficient change, with the y-axis representing the coefficient value and the upper x-axis representing the number of non-zero coefficients in the model at this time; Right: LASSO logic coefficient penalty graph; the first dotted line indicates the minimum value of mean squared error; the second dashed line indicates the position of the lowest point with one standard deviation, indicating the simplest model that can be obtained at the expense of one standard deviation. B: Error rate (Left) and accuracy (Right) of support vector machine model; C: venn diagram of overlapped genes selected by LASSO model and support vector machine model.

The results of LASSO regression showed that 7 genes were characteristic genes, including CPT1B, GATM, LYPLAL1, MGST1, PDK4, SDSL, and TOMM40 (Fig. 3A). The results of SVM-RFE showed that the model achieved the highest accuracy after incorporating the first 9 characteristic genes, including MGST1, TOMM40, PDK4, GATM, MFN2, FASN, LYPLAL1, OXCT1, and CPT1B (Fig. 3B). Finally, 6 genes were demonstrated as genes at the intersection point of Lasso regression and SVM-RFE, including *PDK4, MGST1, TOMM40, LYPLAL1, GATM*, and *CPT1B* (Fig. 3C).

### 3.4. Diagnostic scoring model showed high predictive activity

**Figure 4.**
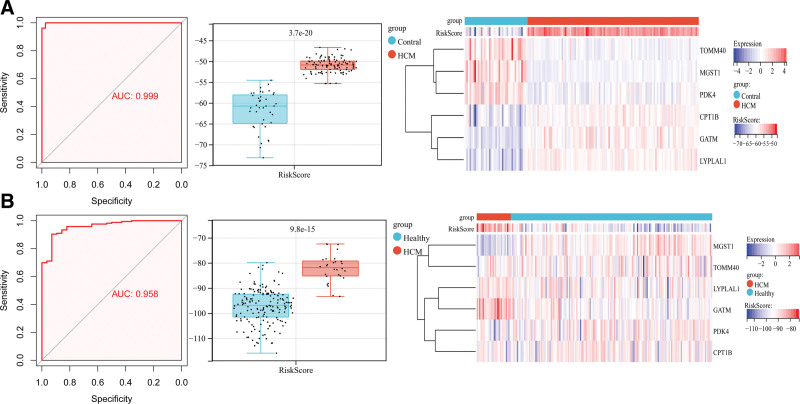
Verification and evaluation of diagnostic model scoring performance. A: verification and evaluation of diagnostic model scoring performance based on data from GSE36961; from left to right: Receiver operating characteristic curve, distribution box diagram of RiskScore in control and hypertrophic cardiomyopathy (HCM) groups, and heatmap of 6 model related genes in each sample; B: verification and evaluation of diagnostic model scoring performance based on data from GSE141910; from left to right: Receiver operating characteristic curve, distribution box diagram of RiskScore in control and hypertrophic cardiomyopathy (HCM) groups, and heatmap of 6 model related genes in each sample.

As shown in Figure 4A, the AUC of the RiskScore model ROC was 0.999, suggesting a valuable predictive role of the model in the training dataset. Meanwhile, the RiskScore of the HCM group was significantly higher than that of the control group (*P* = 3.7e-20). Moreover, the heatmap also showed that the expression levels of the 6 characteristic genes in HCM were significantly different from those in the control group.

Similarly, Figure 4B shows high predictive activity of the RiskScore model in the validation dataset (AUC = 0.958). We observed a significantly higher risk score in patients with HCM than in healthy controls.

### 3.5. Six selected DMEGs-based PPI network and immunological related analysis

**Figure 5.**
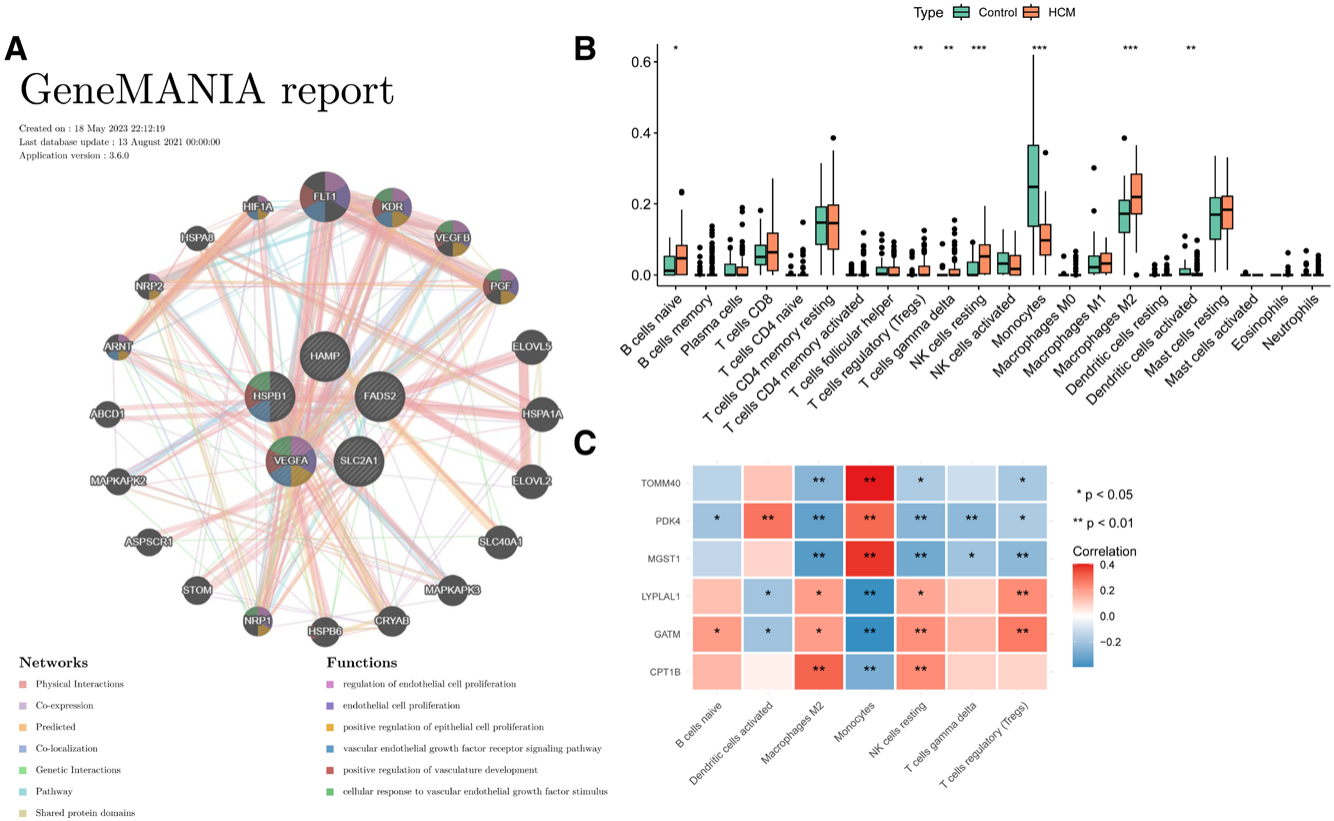
Protein-protein interaction (PPI) network and immunological correlation analysis. A: The PPI network between diagnostic model based differentially expressed mitochondrial genes (DEMGs) and their TOP20 interacting genes; B: The difference on immune cells between hypertrophic cardiomyopathy (HCM) and controls; *versus control, .01 < *P* value < .05, ** versus control, .001 < *P* value < .01, *** versus control, *P* value < .001; C: Correlation analysis between diagnostic genes and immune cells with differential levels in HCM and controls.

A PPI network of the 6 optimal DMEGs and related 20 genes is shown in Figure 5A. The results showed that these genes exhibit co-localization and co-expression relationships.

Immunological analysis showed that the infiltration process of 7 immune cells including monocytes, resting NK cells, and macrophages M2 in HCM was different from that in control, (all *P* < .001, Fig. 5B). The correlation between diagnostic genes and the 7 immune cells was further analyzed. We observed that macrophages M2 and resting NK cells positively correlated with the expression levels of LYPLAL1, GATM, and CPT1B, and negatively correlated with the expression levels of TOMM40, PDK4, and MGST1. Furthermore, monocytes negatively correlated with the expression levels of LYPLAL1, GATM, and CPT1B, and positively correlated with those of TOMM40, PDK4, and MGST1 (Fig. 5C).

### 3.6. Six selected DMEGs-based subtypes

**Figure 6.**
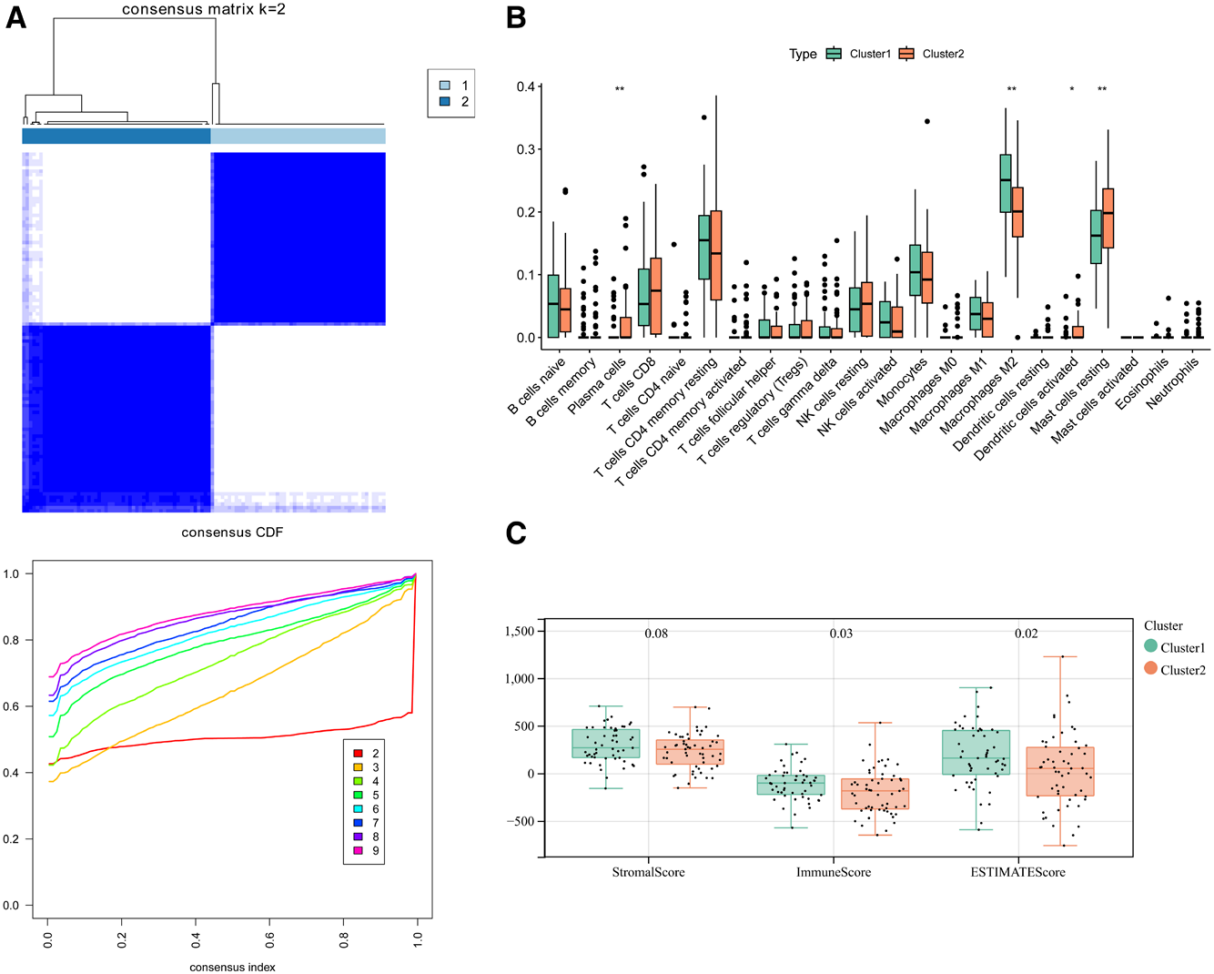
Mitochondrial related molecular subtypes predication and immune cell differences between subtypes. A: Consistency clustering result; B: The difference on immune cells between hypertrophic cardiomyopathy (HCM) and controls; *versus control, .01 < *P* value < .05, ** versus control, .001 < *P* value < .01, *** versus control, *P* value < .001; C: Differences in immune score, matrix score, and ESTIMATEScore between cluster 1 and cluster 2; *versus cluster 1, .01 < *P* value < .05, ** versus cluster 1, .001 < *P* value < .01, *** versus cluster 1, *P* value < .001.

When k = 2, the category in the heatmap was the cleanest and displayed the least noise. Thus, the patients were divided into 2 clusters (Fig. 6A). The infiltration of immune cells between the 2 clusters showed that 4 immune cell types were significantly different including resting mast cells, macrophages M2, and plasma cells (Fig. 6B). Meanwhile, patients in cluster 2 had a significantly lower Matrix score and ESTIMATEScore score (Fig. 6C).

### 3.7. Differences between immune checkpoint genes and HLA family genes, and GSEA analysis

**Figure 7.**
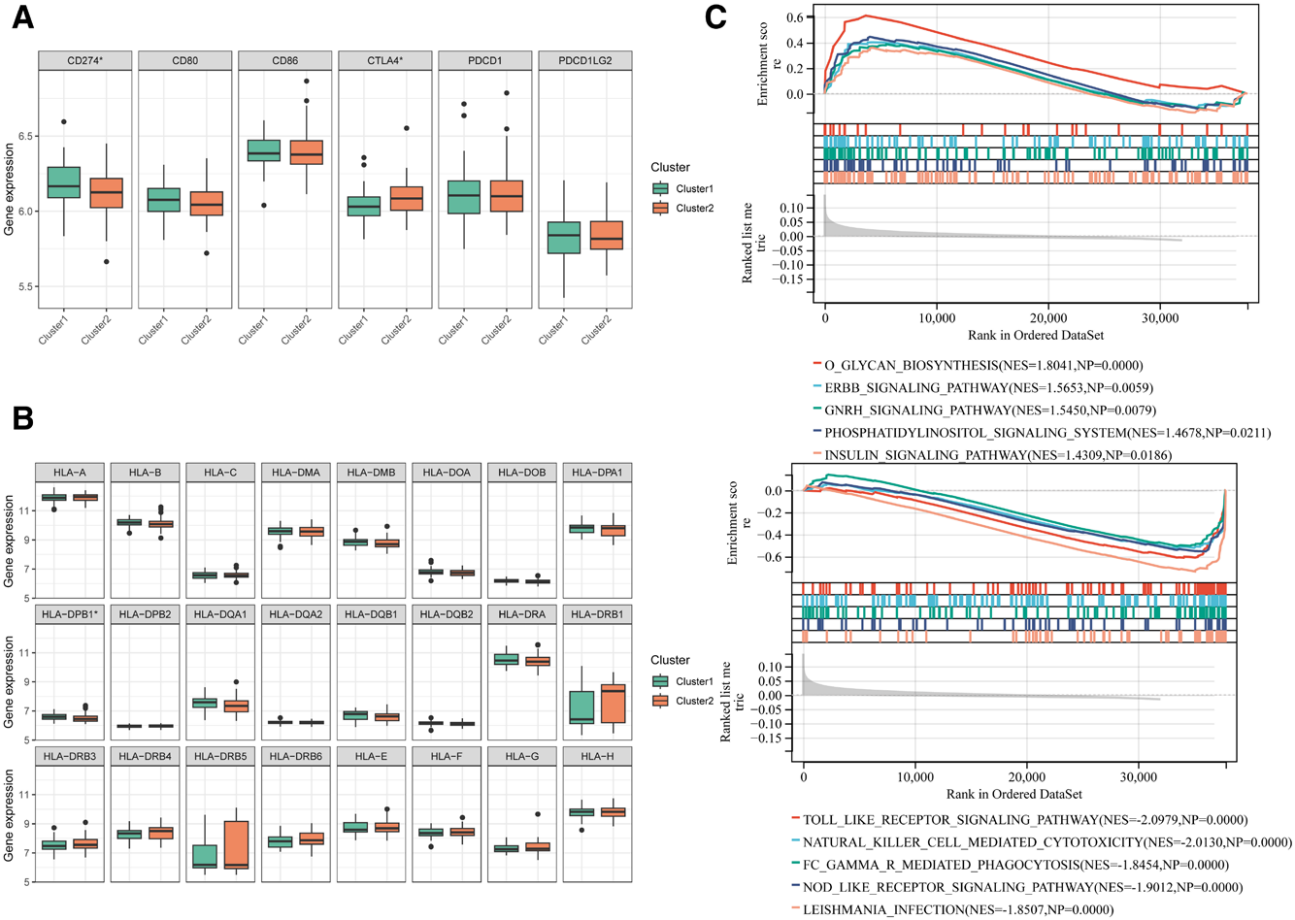
Differences in immune checkpoint genes and HLA family genes between mitochondrial related subtypes. A: Differences in immune checkpoint genes between mitochondrial related subtypes; *versus cluster 1, .01 < *P* value < .05, ** versus cluster 1, .001 < *P* value < .01, *** versus cluster 1, *P* value < .001; B: Differences in HLA family genes between mitochondrial related subtypes; C: Top 5 KEGG pathways based on GSEA; left: up-regulated KEGG pathways, right: down- regulated KEGG pathways.

As shown in Figure 7A, patients in cluster 1 showed higher CD274 levels and lower CTLA4 levels. Regarding differences in HLA family genes, patients in cluster 1 had significantly higher levels of HLA-DPB1 than those in cluster 2 (Fig. 7B).

GSEA analysis related to the KEGG pathway was further performed (Fig. 7C). When we compared cluster 1 with cluster 2, they both were enriched in 9 up-regulated KEGG pathways, including O-glycan biosynthesis (NES = 1.804, NP = 0.0000), ErbB signaling pathway (NES = 1.563, NP = 0.0059), GnRH signaling pathway (NES = 1.804, NP = 0.0000), and 49 other down-regulated KEGG pathways including toll-like signaling pathway, natural killer cell mediated cytotoxicity (NES = 2.079, NP = 0.0000), and Fc gamma R- mediated phagocytosis (NES = 1.8454, NP = 0.0000).

### 3.8. mRNA- transcription factor (TF)-miRNA network

**Figure 8.**
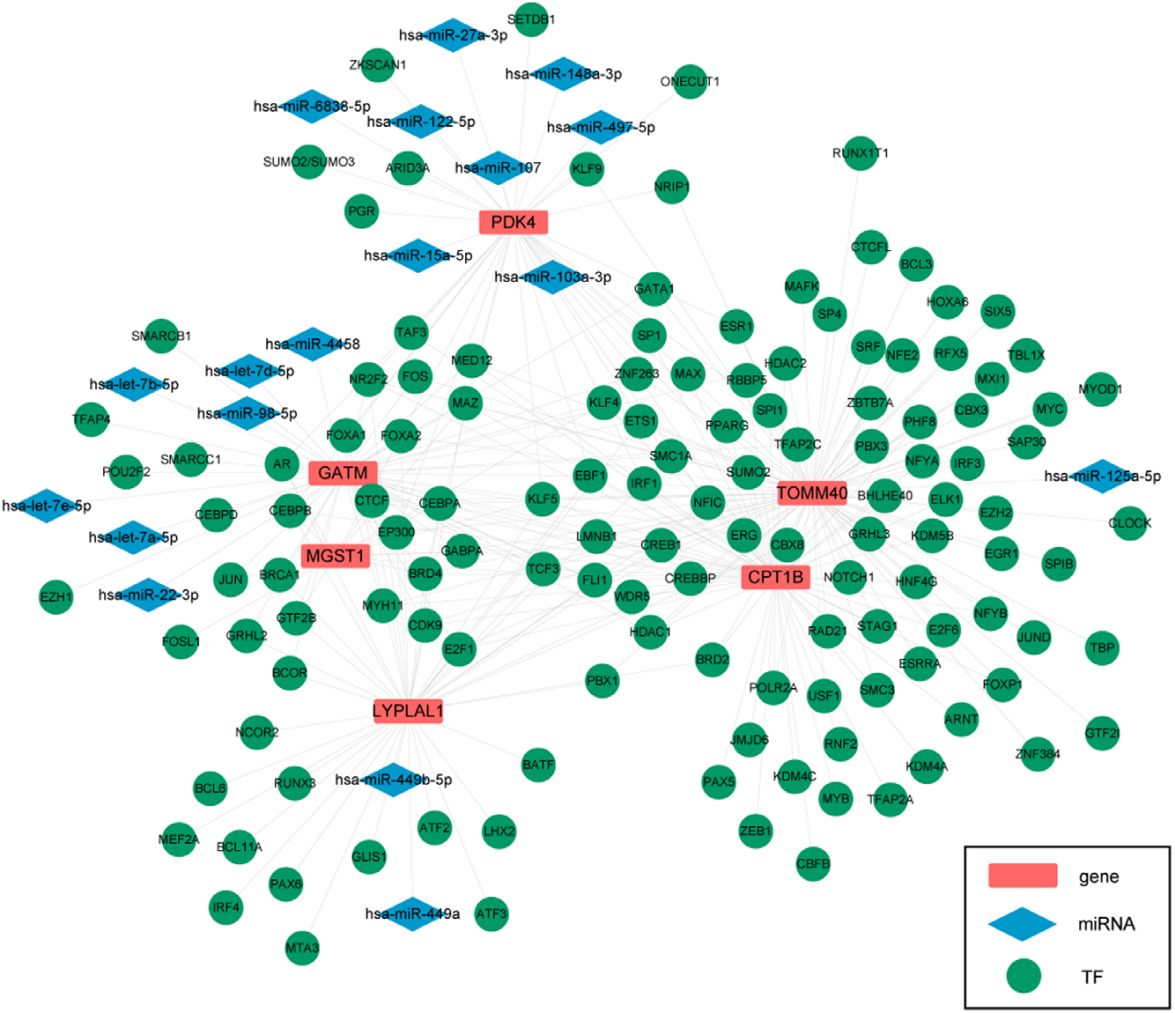
Transcriptional regulatory network. Rectangle represents diagnostic genes, circle represents TF, and diamond represents miRNA. TF = transcription factor.

Next, the related TFs and miRNAs were predicted. Finally, 133 TFs, 18 miRNAs, and 6 mRNAs were identified, which contained 296 TF/miRNA targets (Fig. 8).

## 4. Discussion

Recently, increasing evidence has suggested that HCM is closely related to mutations in mtDNA and inflammatory responses induced by mtDNA. In the present study, 6 DEMGs, including *PDK4, MGST1, TOMM40, LYPLAL1, GATM,* and *CPT1B* were identified as valuable diagnostic biomarkers for HCM. Two clusters were divided based on expression levels of the 6 DEMGs. Different levels of tumor purity and 4 immune cell types were observed between the 2 clusters, including resting mast cell, macrophages M2, and plasma cells. Nine upregulated KEGG pathways were enriched in cluster 1 versus cluster 2, including O-glycan biosynthesis, the ErbB signaling pathway, and GnRH signaling pathway. Meanwhile, 49 other down-regulated pathways were enriched, including the Toll-like signaling pathway and natural killer cell-mediated cytotoxicity pathway.

Growing evidence has confirmed the crucial role of mitochondria in normal functioning of the heart. Mitochondrial dysfunction is highly speculated to cause various cardiac diseases such as HCM and ischemic heart diseases. In the present study, *PDK4, MGST1, TOMM40, LYPLAL1, GATM,* and *CPT1B* were identified as valuable diagnostic biomarkers. Among these 6 mtDNAs, CPT1 is involved in cardiac function by acting as a major energy source, and its activity is recognized as a rate modulator for oxidation of fatty acids.^[27]^ In a mouse model, Haynie et al^[28]^ verified the role of CPT1b in exacerbated cardiac hypertrophy and remodeling. PDK4 plays a role in regulating mitochondrial dynamics, which can prevent diastolic dysfunction.^[29]^ PDK4 upregulation is associated with mitochondrial damage by affecting the status of cellular respiratory system.^[30]^ Mitochondrial dysfunction is a common pathogenic mechanism in patients with HCM, suggesting that it may be important in the pathogenesis of HCM. Although the role of TOMM40 in HCM has not been reported, TOMM40 is a candidate gene that causes mitochondrial dysfunction in late-onset Alzheimer’s disease.^[31]^ Moreover, in the mRNA-TF-miRNA network, TOMM40 was associated with the regulation of multiple genes, suggesting a relatively important role of this gene in HCM. MGST1, as a type of membrane-bound transferase located in mitochondria, mainly participates in cell defense.^[32]^ *LYPLAL1* is significantly associated with fat distribution and GATM plays a role in creatine biosynthesis. Guglielmi *et al* showed that central adiposity could affect regional cardiac hypertrophy in patients with HCM.^[33]^ For the first time, to the best of our knowledge, our data shows links between HCM and genes including MGST1, *LYPLAL1, and* GATM, which may provide a new way to improve our understanding of HCM development.

Furthermore, we showed that these genes were significantly enriched in pathways such as the ErbB signaling pathway, GnRH signaling pathway, and toll-like signaling pathway. The important role of the ErbB family in heart morphogenesis through interaction with the extracellular environment has been well demonstrated.^[34]^ Previous studies have shown that the GnRH pathway is involved in the development of cardiomyopathies.^[35]^ Toll-like receptors, as key factors that regulate innate immune responses, can lead to acute and chronic inflammation.^[36]^ Inflammation is a characteristic feature of cardiac hypertrophy and mediates the important roles of toll-like receptors in the HCM process. Further interventions using molecules involved in these pathways might be of great significance in improving the development of HCM.

It is now widely accepted that recruitment of immune cells to the internal microenvironment precedes hypertrophy and remodeling. This process is accompanied by the modulation of cardiomyocyte and non-cardiomyocyte responses, leading to cardiac failure or dysfunction. Thus, the distribution of immune cell can be used as a biomarker for evaluating cardiac function in HCM. In the present study, we showed that resting mast cells, macrophages M2, and plasma cells may affect the severity of HCM. The heart contains a heterogeneous population of macrophages that participate in cardiac hypertrophy and remodeling regulation.^[37]^ Cardiac macrophage populations are observed before the occurrence of hypertrophy and fibrosis. Once cardiac injury occurs, the number of mast cells increases significantly.^[38]^ IL-4 secreted by mast cells may mediate immunomodulatory and pro-fibrotic effects, and reduced cardiac hypertrophy is usually accompanied by mast cell deficiency. Plasma cells are the final effectors of the B cell lineage; however, their role in HCM has not yet been explored. Taken together, it is important to fully investigate the roles of resting mast cells and macrophages M2 in modulating the function of cardiomyocyte during cardiac remodeling.

Increasing evidence supports the significant contribution of mtDNA to HCM. We demonstrated that a signature based on 6 DEMGs, including *PDK4, MGST1, TOMM40, LYPLAL1, GATM,* and *CPT1B* may be valuable for diagnosis of HCM. Resting mast cells and macrophages M2 may also serve as predictive biomarkers for improving the prognosis of HCM.

## Author contributions

**Conceptualization:** Xuewen Liao, Yazhou Lin.

**Data curation:** Dehua Zeng, Wenming Ying.

**Formal analysis:** Xuewen Liao, Duohuang Lian.

**Investigation:** Xuewen Liao, Meiqing Zhang.

**Methodology:** Dehua Zeng, Meiqing Zhang, Mengmeng Chen.

**Project administration:** Jianjun Ge.

**Resources:** Shunkai Zhou, Duohuang Lian.

**Software:** Wenming Ying, Jianjun Ge.

**Supervision:** Yazhou Lin.

**Validation:** Shunkai Zhou, Yaming Liu.

**Visualization:** Mengmeng Chen, Yaming Liu.

**Writing – original draft:** Xuewen Liao, Dehua Zeng.

**Writing – review & editing:** Xuewen Liao.
